# Utilization patterns and factors associated with persistence of new users of anti-osteoporosis treatment in Denmark: a population-based cohort study

**DOI:** 10.1007/s11657-023-01210-4

**Published:** 2023-01-11

**Authors:** Alma B. Pedersen, Nickolaj Risbo, George Kafatos, David Neasham, James O’Kelly, Vera Ehrenstein

**Affiliations:** 1 Department of Clinical Epidemiology, Aarhus University and Aarhus University Hospital, Olof Palmes Allé 43-45, DK-8200 Aarhus N, Denmark; 2 Amgen Ltd., Cambridge, UK

**Keywords:** Adherence, Alendronate, Denosumab, Osteoporosis, Persistence

## Abstract

***Summary*:**

Persistence with initial treatment was highest after 1 year, decreasing afterwards. Persistence was highest for denosumab followed by alendronate. We identified several factors associated with treatment persistence, some of which were the same irrespective of OTx agent, which could help target subgroups of patients in terms of social and healthcare support.

**Purpose:**

To describe patient characteristics, persistence, and factors associated with the persistence of new users of the bisphosphonates (alendronate, risedronate, and ibandronate) and the RANKL inhibitor denosumab in Denmark.

**Methods:**

A population-based cohort study using health registries (2010–2018). We included alendronate (*n* = 128,590), risedronate (*n* = 892), ibandronate (*n* = 5,855), and denosumab (*n* = 16,469) users, aged ≥ 50 years.

**Results:**

The 1-year persistence was 68.2% in the alendronate cohort; 39.3% in the risedronate cohort; 56.3% in the ibandronate cohort; and 84.0% in the denosumab cohort. The 2-year persistence was 58.7% in the alendronate cohort; 28.0% in the risedronate cohort; 42.9% in the ibandronate cohort; and 71.9% in the denosumab cohort. The 4-year persistence was 46.3%, 15.4%, 29.6%, and 56.9%, respectively. Later years of treatment initiation were associated with lower persistence for alendronate (adjusted odds ratio (OR) with 95% CI was 0.86 (0.81–0.91) in 2016 compared to 2010), but not for risedronate (OR was 1.56 (0.60–4.06), ibandronate (OR was 0.92 (0.71–1.19) or denosumab (OR was 1.11 (0.87–1.43). Older age was associated with higher persistence for all medications and the same goes for the female sex except for ibandronate. Dementia was associated with higher persistence for alendronate but not denosumab, whereas prior osteoporosis treatment (OT) was the opposite. Several comorbidities were associated with lower persistence for alendronate, but not denosumab.

**Conclusion:**

Persistence was highest for denosumab followed by alendronate. We identified several factors associated with treatment persistence, some of which were the same irrespective of OTx agent, which could help target subgroups of patients in terms of social and healthcare support.

**Supplementary Information:**

The online version contains supplementary material available at 10.1007/s11657-023-01210-4.

## Introduction

Osteoporosis affects 1 in 3 postmenopausal women and 1 in 5 men older than 50 years [1]. In Denmark, anti-osteoporosis treatments spanning multiple classes have been used extensively, [2] with the bisphosphonate alendronate being the first-line and most commonly used agent, in accordance with the Danish treatment guidelines [3]. Other bisphosphonates may be also used, including risedronate and ibandronate. In addition, the monoclonal antibody directed against RANK-ligand, denosumab, has been introduced in 2010 [4]. Typically, treatments other than alendronate are used second-line as they may be prescribed if alendronate is not well-tolerated or in case of treatment failure or under special circumstances such as impaired renal function [3]. Some evidence suggests that the RANKL inhibitor denosumab may be a favored second-line treatment in Denmark compared with the bisphosphonates risedronate and ibandronate [5]. 

Poor adherence with osteoporosis treatment is well documented [6–9], and predictors of poor persistence and refill compliance have been investigated previously; however, results are inconsistent [10–12]. Poor adherence remains a significant clinical concern as it may impact both the risk of incident and imminent fractures [13] and further insight into this issue is warranted. Using the Danish nationwide population-based registries allows for population-wide studies, minimizing selection bias. In addition, persistence to denosumab treatment has not been previously described in Denmark. A better understanding of any potential predictors of persistence [14, 15] will help inform clinical practice and may guide the optimal use of these therapies, especially given the “treatment gap,” identified in several studies of patients with osteoporosis [14, 16–18]. 

We conducted a population-based cohort study to describe characteristics of new users of the bisphosphonates alendronate, risedronate, and ibandronate and the RANKL inhibitor denosumab in Denmark. Among them, we assessed persistence with the initial treatment agent and factors associated with persistence.

## Methods

### Setting

This study was based on routinely collected health and administrative data, linked from several population-based registries in Denmark in a setting of universal health care, complete lifetime follow-up, and individual-level linkage [19].

### Data sources

The following registries were used to extract data for this study:

The Danish National Prescription Registry [20, 21], which holds information related to all outpatient dispensing of prescribed drugs dating back to 1995. For the current study, dates of sale, the anatomical therapeutic chemical (ATC) codes, and the amount dispensed were extracted. The Danish Civil Registration System [22, 23], which tracks residence and vital status for Danish residents since 1968, assigns the unique personal identifier. For the current study, information on (pseudonymized) identifier, birth date, residence, vital status, and migration status was extracted. The Danish National Patient Registry [24] captures information on all hospital encounters including inpatient stays since 1977, as well as outpatient specialist clinic visits and emergency encounters since 1995. For the current study, information on the date of contact, discharge diagnoses, and radiological procedures was extracted. Integrated Database for Labour Market Research (IDLMR) [25] collects socioeconomic information on all residents in Denmark since 1980. For the current study, information on education, income, and employment status was extracted.

### Study population

The inclusion criteria were residence in Denmark and initiation of alendronate, risedronate, ibandronate, or denosumab in osteoporosis doses between 26 May 2010 (date of denosumab approval by the European Medicines Agency [26]) and 31 December 2018 (the study period). In Denmark, the dosages and dose intervals used for the treatment of osteoporosis are 70 mg once weekly, 150 mg once monthly, 35 mg once weekly, and 60 mg every 6 months for alendronate, ibandronate, risedronate, and denosumab, respectively. The initiators were identified based on outpatient dispensing recorded in the Danish National Prescription Registry. Patients were enrolled in the alendronate cohort based on the first dispensing of alendronate in the study period. Likewise, patients were enrolled in the risedronate cohort based on a first dispensing of risedronate in the study period, in the ibandronate cohort based on a first dispensing of ibandronate in the study period, and in the denosumab cohort based on a first dispensing of denosumab in the study period. The first dispensing during the study period defined the start of the follow-up (the index date). The exclusion criteria were age younger than 50 years on the index date, a dispensing of the index anti-osteoporosis agent before 26 May 2010, a diagnosis of Paget’s disease any time before the index date, and a dispensing of more than one anti-osteoporosis agent on the index date.

### Persistence

In the main analyses, a 2-year persistence with each of the four medications was examined in the subset of patients with the opportunity for 2 years of follow-up. Coverage of each dispensing was estimated based on the number of defined daily doses supplied based on the number of packages and package size in each dispensing. Once the duration of each dispensing is estimated, the initial continuous treatment episodes were established by adding a 60-day grace period to the computed duration end. Treatment persistence was expressed as a cumulative incidence of non-persistence. Non-persistence occurred on the date after the end of initial continuous treatment episode ended without a new dispensing (grace period) or when the patient had a dispensing of another antiresorptive agent (including alendronate, risedronate, ibandronate, or denosumab). Persons who were censored due to death, emigration, Paget’s disease, or malignancy during the initial continuous treatment episode were considered persistent. Two-year persistence was the primary outcome in the population with the appropriate follow-up opportunity. One- and 4-year persistence was examined as secondary outcomes in patients with the appropriate follow-up.

### Covariates

Factors potentially associated with treatment persistence were assessed among the patients’ characteristics measured over different lookback periods before initiation of the index treatment. On the index date: calendar year, age, sex, area of residence, highest achieved education, employment status. In the year before the index date: concomitant medication (one year before the index date), annual household income (in the 5 calendar years prior to the index year as the mean annual household income), healthcare resource utilization (number of hospitalizations, outpatient/emergency visits 1 year before the index date).

Any time before the index date back to the start of the Danish National Prescription Registry based on the ATC codes recording of variables: prior dispensing of osteoporosis treatment (OTx) and time since last prior dispensing of OTx. Any time before the index date back to the start of the Danish National Patient Registry based on the ICD codes (and in case of chronic obstructive pulmonary disease and diabetes based on ATC codes too) recording of variables: major osteoporotic fractures (hip, clinical vertebral, humerus, and wrist fracture), time since last major osteoporotic fracture, hospital diagnosis of osteoporosis, time since diagnosis of osteoporosis, the measured comorbidities, including the Charlson comorbidity index [27], dual-energy X-ray absorptiometry (DXA) procedure, time since last DXA procedure, other specific comorbidity diagnoses such as chronic obstructive pulmonary disease, chronic renal impairment, diabetes, and dementia. ATC and ICD codes used in this study are presented in Supplementary Table 3.

### Statistical analyses

The baseline characteristics of the four exposure cohorts including all patients and after excluding patients with a history of malignancy were described overall using descriptive statistics. Persistence was examined in treatment initiators without a history of malignancy to exclude the use of the agents for cancer indication and thus in different dosages and durations. For the persistence, cumulative incidence curves of persistence with death as a competing risk were estimated and plotted for each exposure cohort. Time for the individual person was censored at emigration, a diagnosis of Paget’s disease, malignancy, or end of the study, should any of these happen before non-persistence. Estimates of persistence at 2 (and 1 and 4) years in the patient subpopulations with the possibility to follow-up of 2 (1 and 4) years were read off from the cumulative incidence curves of persistence and reported as proportions with 95% confidence intervals (CIs).

The association between covariates and treatment persistence was evaluated using logistic regression at 2 (and 1 and 4) years on complete case data (excluding observations with missing values). We used all measured baseline variables with a sufficient number of outcomes (more than 5) as potential factors of association and computing crude and multivariable odds ratios (ORs) and associated 95% CIs. We focused on both point estimates and their precision to highlight specific factors associated with persistence. Analyses were performed separately for each of the four drugs since clinical indication, line of treatment, and selection of patients for treatment are rather different for the three oral bisphosphonates and denosumab, and thus, most likely, the factors associated with persistence may also be different.

Sensitivity analyses were performed by evaluating 2-year persistence using grace periods of 30, 90, and 120 days.

All analyses were performed in SAS statistical software. The study is reported according to STROBE guidelines.

## Results

From 2010 through 2018, we included 128,590 patients in the alendronate initiators cohort, 892 patients in the risedronate initiators cohort, 5855 patients in the ibandronate initiator cohort, and 16,469 patients in the denosumab initiators cohort. Table 1 shows the baseline characteristics of the four cohorts. Males comprised 21.4% of the alendronate cohort, 13.3% of the risedronate cohort, 11.4% of the ibandronate cohort, and 11.0% of the denosumab cohort. The median (quartiles) patient age was 70 (63–78) in the alendronate cohort, 70 (63–77) years in the risedronate and ibandronate cohorts, and 72 (65–80) years in the denosumab cohort. The proportion of patients included in each of the cohorts slightly decreased over the duration of the study period. Educational level was similar in all four cohorts. Compared to the bisphosphonate cohorts, the denosumab cohort appeared to have a somewhat higher prevalence of early retirement, higher Charlson comorbidity index, chronic obstructive pulmonary disease, chronic renal impairment, dementia, malignancy, hospital-diagnosed osteoporosis, history of fracture, prior OTx, the use of concomitant medication such as hormone replacement therapy, hormone deprivation therapy, anxiolytics, and sedatives and had a longer median time from diagnosis of last major osteoporosis fracture until treatment initiation. The use of corticosteroids, antidiabetics, and non-steroid anti-inflammatory drugs appeared to be lower in the denosumab cohort than in the bisphosphonates cohorts. The descriptive data suggest regional prescribing preferences for an OTx agent. Regarding healthcare utilization in the year before the index date, it seems that the denosumab cohort had a higher prevalence of patients with no hospitalizations and no emergency room visits, but a slightly lower prevalence of patients with no outpatient visits (Table 1). Baseline characteristics of the four cohorts restricted to patients without a history of malignancy are presented in Supplementary Table 1.

**Table 1:** Baseline Characteristics of new users of alendronate, risedronate, ibandronate, and denosumab

	Alendronate	Risedronate	Ibandronate	Denosumab
	*N* = 128,590	*N* = 892	*N* = 5855	*N* = 16,469
Sex, *n* (%)				
Female	101,119 (78.6)	773 (86.7)	5186 (88.6)	14,659 (89.0)
Male	27,471 (21.4)	119 (13.3)	669 (11.4)	1810 (11.0)
Age, years median (IQR)	70 (63–78)	70 (63–77)	70 (63–77)	72 (65–80)
Age group, *n* (%)				
50– < 55	7056 (5.5)	51 (5.7)	271 (4.6)	569 (3.5)
55– < 60	12,419 (9.7)	90 (10.1)	598 (10.2)	1192 (7.2)
60– < 65	17,402 (13.5)	116 (13.0)	816 (13.9)	1885 (11.4)
65– < 70	22,738 (17.7)	173 (19.4)	1075 (18.4)	2843 (17.3)
70– < 75	22,742 (17.7)	167 (18.7)	1093 (18.7)	2984 (18.1)
75 +	46,233 (36.0)	295 (33.1)	2002 (34.2)	6996 (42.5)
Year of treatment initiation, *n* (%)				
2010	9514 (7.4)	53 (5.9)	668 (11.4)	490 (3.0)
2011	16,866 (13.1)	209 (23.4)	949 (16.2)	2253 (13.7)
2012	15,882 (12.4)	113 (12.7)	640 (10.9)	2241 (13.6)
2013	15,237 (11.8)	76 (8.5)	638 (10.9)	2037 (12.4)
2014	14,699 (11.4)	115 (12.9)	654 (11.2)	2128 (12.9)
2015	14,435 (11.2)	43 (4.8)	623 (10.6)	2084 (12.7)
2016	14,255 (11.1)	76 (8.5)	567 (9.7)	1819 (11.0)
2017	13,895 (10.8)	96 (10.8)	528 (9.0)	1737 (10.5)
2018	13,807 (10.7)	111 (12.4)	588 (10.0)	1680 (10.2)
Region of residence, *n* (%)				
Capital	31,725 (24.7)	283 (31.7)	1356 (23.2)	4049 (24.6)
Zealand	18,404 (14.3)	90 (10.1)	1144 (19.5)	1962 (11.9)
Southern Denmark	30,335 (23.6)	182 (20.4)	1038 (17.7)	2865 (17.4)
Central Jutland	31,786 (24.7)	222 (24.9)	1532 (26.2)	5152 (31.3)
Northern Jutland	16,340 (12.7)	115 (12.9)	785 (13.4)	2441 (14.8)
Annual household income, *n* (%)				
< 200,000 kr	21,694 (16.9)	150 (16.8)	945 (16.1)	2911 (17.7)
200,000–300,000 kr	36,927 (28.7)	237 (26.6)	1706 (29.1)	5029 (30.5)
300,000–400,000 kr	22,596 (17.6)	153 (17.2)	1050 (17.9)	2939 (17.8)
≥ 400,000 kr	47,252 (36.7)	352 (39.5)	2154 (36.8)	5590 (33.9)
Missing	121 (0.1)	↑	↑	↑
Educational level, *n* (%)				
Primary school	53,004 (41.2)	327 (36.7)	2375 (40.6)	6850 (41.6)
Secondary school	49,131 (38.2)	345 (38.7)	2295 (39.2)	6026 (36.6)
Higher	23,114 (18.0)	202 (22.6)	1036 (17.7)	3178 (19.3)
Missing	3341 (2.6)	18 (2.0)	149 (2.5)	415 (2.5)
Employment status, *n* (%)				
Director/chief executive	10,177 (7.9)	78 (8.7)	410 (7.0)	956 (5.8)
Employer/self-employed	3366 (2.6)	22 (2.5)	150 (2.6)	342 (2.1)
Skilled worker	8873 (6.9)	47 (5.3)	339 (5.8)	671 (4.1)
Unskilled worker	2013 (1.6)	12 (1.3)	74 (1.3)	134 (0.8)
Early retirement/pension	97,371 (75.7)	693 (77.7)	4559 (77.9)	13,727 (83.4)
Unemployed, benefits/public support	2988 (2.3)	12 (1.3)	123 (2.1)	217 (1.3)
Other	3802 (3.0)	28 (3.1)	200 (3.4)	422 (2.6)
Missing	↑	0 (0.0)	0 (0.0)	0 (0.0)
Comorbidities				
Charlson comorbidity index (CCI), *n* (%)				
Low	78,363 (60.9)	545 (61.1)	3736 (63.8)	9584 (58.2)
Medium	39,427 (30.7)	274 (30.7)	1692 (28.9)	5215 (31.7)
High	10,800 (8.4)	73 (8.2)	427 (7.3)	1670 (10.1)
Individual comorbidities, *n* (%)				
Chronic obstructive pulmonary disease	15,719 (12.2)	98 (11.0)	722 (12.3)	2241 (13.6)
Chronic renal impairment	1679 (1.3)	13 (1.5)	31 (0.5)	478 (2.9)
Diabetes	11,836 (9.2)	67 (7.5)	399 (6.8)	1327 (8.1)
Dementia	2395 (1.9)	8 (0.9)	83 (1.4)	396 (2.4)
Any malignancy	22,326 (17.4)	145 (16.3)	954 (16.3)	3043 (18.5)
Breast cancer	7951 (6.2)	48 (5.4)	399 (6.8)	1063 (6.5)
Prostate cancer	1778 (1.4)	< 5	33 (0.6)	185 (1.1)
Intestine cancer	2708 (2.1)	17 (1.9)	117 (2.0)	365 (2.2)
Lung cancer	1583 (1.2)	9 (1.0)	66 (1.1)	200 (1.2)
Pancreatic cancers	118 (0.1)	0 (0.0)	< 5	25 (0.2)
Cancer of the urinary tract, including kidneys	877 (0.7)	6 (0.7)	26 (0.4)	127 (0.8)
Hematological malignancy	1808 (1.4)	20 (2.2)	62 (1.1)	228 (1.4)
Metastasis and non-specified cancer in lymph nodes	1911 (1.5)	9 (1.0)	86 (1.5)	266 (1.6)
Other malignancy	7722 (6.0)	56 (6.3)	330 (5.6)	1188 (7.2)
Osteoporosis-related characteristics				
Hospital diagnosis of osteoporosis, *n* (%)	48,556 (37.8)	484 (54.3)	2520 (43.0)	11,931 (72.4)
Months from hospital diagnosis of osteoporosis to index date among patients with hospital diagnosis of osteoporosis, median (IQR)	2 (0–7)	19 (5–56)	22 (5–61)	31 (7–79)
History of hip fracture, *n* (%)	11,814 (9.2)	50 (5.6)	382 (6.5)	1740 (10.6)
History of vertebral fracture, *n* (%)	4136 (3.2)	22 (2.5)	186 (3.2)	880 (5.3)
History of forearm fracture, *n* (%)	18,906 (14.7)	152 (17.0)	917 (15.7)	3143 (19.1)
History of humerus fracture, *n* (%)	7880 (6.1)	52 (5.8)	393 (6.7)	1362 (8.3)
Months from diagnosis of last major osteoporotic fracture to index date among patients with hospital diagnosis of hip, vertebral, forearm, or humerus fracture, median (IQR)	53 (7–133)	67 (22–150)	71 (22–145)	80 (28–149)
DXA scan procedure, *n* (%)	100,178 (77.9)	777 (87.1)	5143 (87.8)	15,215 (92.4)
Months from the last DXA procedure to the index date among patients with DXA procedure, median (IQR)	2 (0–34)	28 (5–66)	25 (4–68)	50 (13–90)
Prior osteoporosis treatment (OT)x, *n*				
All OTx	5980 (4.7)	754 (84.5)	4727 (80.7)	14,128 (85.8)
Raloxifene	848 (0.7)	16 (1.8)	121 (2.1)	727 (4.4)
Teriparatide	1107 (0.9)	21 (2.4)	87 (1.5)	1060 (6.4)
Parathyroid hormone	108 (0.1)	6 (0.7)	9 (0.2)	159 (1.0)
Etidronate	2646 (2.1)	66 (7.4)	304 (5.2)	1467 (8.9)
Alendronate	0 (0.0)	741 (83.1)	4602 (78.6)	13,217 (80.3)
Ibandronate	851 (0.7)	45 (5.0)	< 5	1854 (11.3)
Risedronate	337 (0.3)	0 (0.0)	91 (1.6)	390 (2.4)
Zoledronic acid	6 (0.0)	< 5	< 5	40 (0.2)
Strontium ranelate	558 (0.4)	24 (2.7)	201 (3.4)	1037 (6.3)
Denosumab	160 (0.1)	30 (3.4)	85 (1.5)	0 (0.0)
Months from the last dispensing of prior OTx to the index date among patients with prior OTx use, *n* (%)	106 (27–151)	14 (4–53)	16 (4–55)	51 (14–101)
Concomitant medication, *n* (%)				
Oral corticosteroids	32,974 (25.6)	191 (21.4)	1208 (20.6)	2719 (16.5)
Anticoagulants	10,999 (8.6)	54 (6.1)	377 (6.4)	1468 (8.9)
Antidiabetics	9172 (7.1)	54 (6.1)	295 (5.0)	938 (5.7)
Antithrombotics	33,477 (26.0)	209 (23.4)	1434 (24.5)	4501 (27.3)
Hormone replacement theory	19,178 (14.9)	178 (20.0)	1149 (19.6)	3273 (19.9)
Hormone deprivation theory	159 (0.1)	0 (0.0)	9 (0.2)	16 (0.1)
Anxiolytics and sedatives	26,790 (20.8)	191 (21.4)	1420 (24.3)	4187 (25.4)
Antipsychotics	4617 (3.6)	25 (2.8)	187 (3.2)	609 (3.7)
Antidepressants	22,788 (17.7)	125 (14.0)	1091 (18.6)	3433 (20.8)
Statins	38,591 (30.0)	232 (26.0)	1603 (27.4)	4827 (29.3)
Non-steroid anti-inflammatory drugs	40,821 (31.7)	243 (27.2)	1725 (29.5)	4131 (25.1)
Antihypersentive drugs	70,983 (55.2)	451 (50.6)	3064 (52.3)	9222 (56.0)
Drugs for the treatment of chronic obstructive pulmonary disease	37,226 (28.9)	278 (31.2)	1864 (31.8)	5080 (30.8)
Opioids	46,855 (36.4)	247 (27.7)	1950 (33.3)	6171 (37.5)
Anti-thyroid drugs	2425 (1.9)	15 (1.7)	133 (2.3)	345 (2.1)
Healthcare utilization in the year before the index date, *n* (%)				
Number of hospitalizations				
0	83,905 (65.3)	678 (76.0)	4453 (76.1)	11,194 (68.0)
1	27,200 (21.2)	154 (17.3)	942 (16.1)	2920 (17.7)
> 1	17,485 (13.6)	60 (6.7)	460 (7.9)	2355 (14.3)
Number of outpatient visits				
0	36,006 (28.0)	249 (27.9)	2100 (35.9)	3190 (19.4)
1	25,674 (20.0)	184 (20.6)	1101 (18.8)	3217 (19.5)
> 1	66,910 (52.0)	459 (51.5)	2654 (45.3)	10,062 (61.1)
Number of emergency room visits				
0	98,700 (76.8)	737 (82.6)	4925 (84.1)	13,140 (79.8)
1	22,629 (17.6)	123 (13.8)	746 (12.7)	2460 (14.9)
> 1	7261 (5.6)	32 (3.6)	184 (3.1)	869 (5.3)

**Fig. 1:**
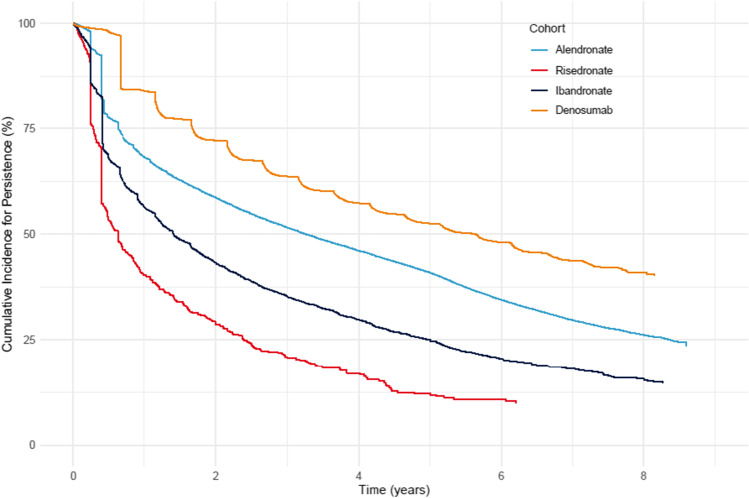
Cumulative incidence curves for persistence by treatment cohort during the follow-up

Table 2 shows 2-year persistence among patients without a history of malignancy at baseline overall and in selected restricted subpopulations. Among patients initiating treatment in 2010–2016, the 2-year persistence was 58.7% (58.4–59.1) in the alendronate cohort, 28.0% (24.5–31.8) in the risedronate cohort, 42.9% (41.3–44.4) in the ibandronate cohort, and 71.9% (71.0–72.7) in the denosumab cohort. The 2-year persistence was higher among female compared to male patients and among patients who were recent osteoporosis treatment users compared to those who were past osteoporosis treatment users for all four OTx agents. Compared to overall 2-year persistence, persistence to alendronate, risedronate, or ibandronate was slightly higher, whereas persistence to denosumab was slightly lower when restricted to patients with a history of hip fracture before the index date. Persistence was similar to overall 2-year persistence among patients with a history of different other fractures before the index date. Figure 1 shows cumulative incidence curves for persistence during the follow-up. The graph shows that persistence decreases consistently across all the cohorts over time, but slightly less for the denosumab cohort during the first 3–6 months due to the nature of the denosumab administration. Results of sensitivity analyses on 2-year persistence using grace periods of 30, 90, and 120 days were consistent with the main analysis.

**Table 2:** Two-year persistence by treatment cohort overall and in selected subgroups among patients without malignancy at baseline initiating treatment in 2010–2016

				2-year persistence
	Treatment cohort	*N* at follow-up start	*n*	% (95% CI)
Overall	Alendronate	85,486	50,749	58.7 (58.4–59.1)
	Risedronate	594	171	28.0 (24.5–31.8)
	Ibandronate	4073	1789	42.9 (41.3–44.4)
	Denosumab	11,054	8007	71.9 (71.0–72.7)
Women	Alendronate	67,697	40,720	59.6 (59.3–60.0)
	Risedronate	523	152	28.3 (24.6–32.4)
	Ibandronate	3617	1585	42.8 (41.2–44.4)
	Denosumab	9911	7220	72.3 (71.5–73.2)
Men	Alendronate	17,789	10,029	55.2 (54.5–55.9)
	Risedronate	71	19	25.5 (16.7–37.7)
	Ibandronate	456	204	43.4 (38.9–48.1)
	Denosumab	1143	787	68.0 (65.2–70.7)
Restricted to recent osteoporosis treatment users	Alendronate	3132	1989	63.0 (61.3–64.7)
	Risedronate	427	120	27.4 (23.4–32.0)
	Ibandronate	2780	1201	42.2 (40.3–44.0)
	Denosumab	6980	5259	74.9 (73.9–75.9)
Restricted to past osteoporosis treatment users	Alendronate	1544	768	49.1 (46.6–51.6)
	Risedronate	60	12	18.6 (10.6–31.6)
	Ibandronate	508	172	32.2 (28.3–36.6)
	Denosumab	2563	1735	66.9 (65.1–68.8)
Restricted to osteoporosis naïve patients	Alendronate	80,810	47,992	58.7 (58.4–59.1)
	Risedronate	107	39	35.4 (27.0–45.5)
	Ibandronate	785	416	52.2 (48.7–55.8)
	Denosumab	1511	1013	66.4 (64.0–68.8)
Restricted to patients with a history of vertebral fracture before the index date	Alendronate	2607	1525	57.8 (55.9–59.7)
	Risedronate	12	5	41.7 (18.8–75.3)
	Ibandronate	118	54	43.5 (34.9–53.3)
	Denosumab	569	416	72.2 (68.4–75.9)
Restricted to patients with a history of hip fracture before the index date	Alendronate	7829	4952	62.5 (61.4–63.6)
	Risedronate	35	14	40.0 (25.7–58.5)
	Ibandronate	268	125	45.0 (39.2–51.3)
	Denosumab	1111	774	68.8 (66.0–71.5)
Restricted to patients with a history of humerus and/or forearm fracture before the index date	Alendronate	16,131	9886	60.7 (60.0–61.5)
	Risedronate	115	40	34.3 (26.3–43.9)
	Ibandronate	806	363	43.8 (40.4–47.4)
	Denosumab	2706	1936	70.9 (69.2–72.7)
Restricted to patients with no history of any major osteoporotic fracture before the index date	Alendronate	62,021	36,380	58.0 (57.6–58.4)
	Risedronate	446	119	25.7 (21.9–30.1)
	Ibandronate	2998	1301	42.4 (40.6–44.2)
	Denosumab	7316	5330	72.4 (71.3–73.4)
Restricted to patients with a history of dxa procedure before the index date	Alendronate	66,079	39,244	58.8 (58.4–59.2)
	Risedronate	513	147	28.0 (24.3–32.1)
	Ibandronate	3544	1552	42.8 (41.2–44.5)
	Denosumab	10,200	7407	72.1 (71.2–73.0)

Among patients without malignancy at baseline initiating treatment in 2010–2017, the 1-year persistence was 68.2% (67.9–68.5) in the alendronate cohort; 39.3% (35.7–43.1) in the risedronate cohort; 56.3% (54.9–57.8) in the ibandronate cohort; and 84.0% (83.4–84.7) in the denosumab cohort. The 4-year persistence was 46.3% (45.9–46.7), 15.4% (12.5–19.0), 29.6% (27.9–31.2), and 56.9% (55.8–58.0), respectively.

**Fig. 2:**
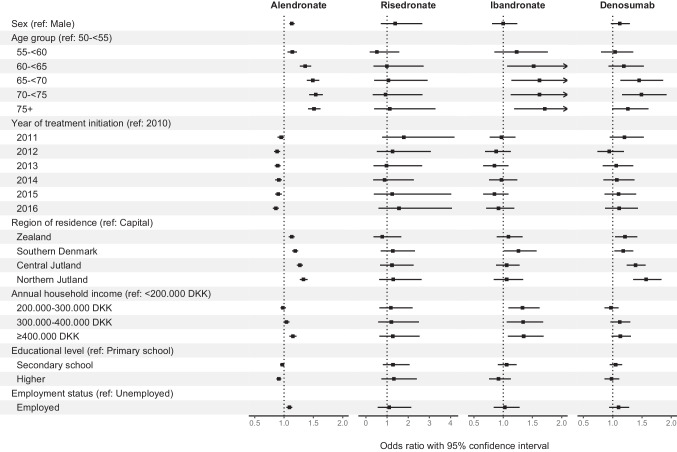
Patient demographics association with persistence after 2 years by treatment cohort among patients without malignancy at baseline initiating treatment in 2010–2016. Please note different scale on *x*-axis for Risedronate compared to the other three treatments

**Fig. 3:**
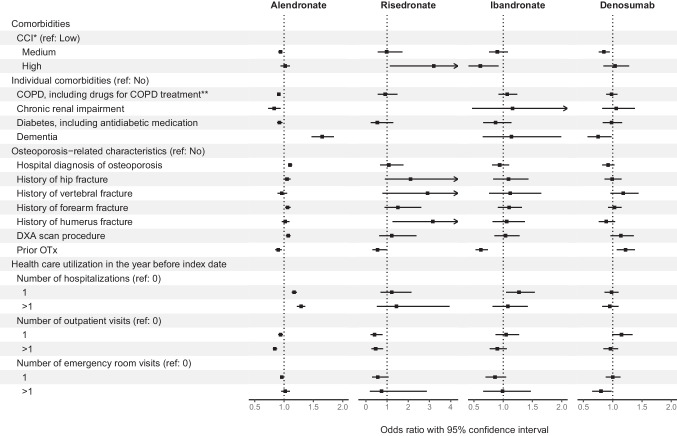
Comorbidities and health care utilization factors associated with persistence after 2 years by treatment cohort among patients without malignancy at baseline initiating treatment in 2010–2016. Please note different scales on *x*-axis for Risedronate compared to the other three treatments. *CCI, Charlson comorbidity index. **COPD, chronic obstructive pulmonary disease

**Fig. 4:**
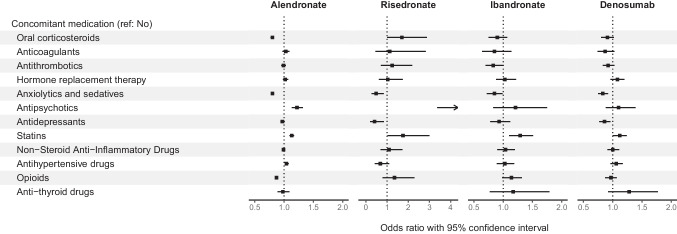
Concomitant medication association with persistence after 2 years by treatment cohort among patients without malignancy at baseline initiating treatment in 2010–2016. Please note different scales on *x*-axis for Risedronate compared to the other three treatments

Figures 2, 3, and 4 show adjusted ORs for the association between the baseline characteristics and 2-year persistence. Results for the risedronate cohort tended to be imprecise due to the small sample size.

For all four OTx agents, 2-year persistence increased for older age groups. Female sex was associated with the persistence of alendronate, risedronate, and denosumab (but not ibandronate). Later years of treatment initiation were associated with lower persistence for alendronate (adjusted Odds ratio (OR) was 0.86 (0.81–0.91) in 2016 compared to 2010), but not for risedronate (OR was 1.56 (0.60–4.06), ibandronate (OR was 0.92 (0.71–1.19) or denosumab (OR was 1.11 (0.87–1.43). Persistence seemed to vary according to the region of residence, being slightly higher in all four regions compared with capital region irrespective of OTx agent. It seemed that higher household income was associated with higher persistence for all OTx agents. The highest educational level seemed to be associated with lower persistence for alendronate and ibandronate, but not risedronate and denosumab. Employment status seemed to be associated with the persistence of alendronate and denosumab. The increasing number of hospitalizations was associated with higher persistence of alendronate, but not denosumab, whereas a number of outpatient and emergency room visits were not associated with persistence to any OT agent.

CCI level was not associated with the 2-year persistence of any OTx agent. On the other hand, individual comorbidities such as COPD, chronic renal impairment, and diabetes were associated with lower persistence of alendronate, but not denosumab. Dementia was associated with higher persistence with alendronate, but lower persistence with denosumab. The history of hip and forearm fractures seemed to be associated with 2-year persistence with alendronate, whereas the history of vertebral fracture seemed to be associated with 2-year persistence with denosumab. The DXA scan procedure before the index date was associated with persistence to alendronate with similar trends for other OTx agents.

Regarding concomitant medication, the use of anxiolytics, sedatives, and opioids was associated with lower persistence of both alendronate and denosumab, whereas the use of statins and antipsychotics was associated with higher persistence. Prior OT use of other agents was associated with lower persistence with alendronate, and higher persistence with denosumab.

## Discussion

In this population-based cohort study of new users of osteoporosis medication, alendronate accounted for most of the initiated treatment, while denosumab constituted the second largest group. Two-year persistence varied by the index OTx agent and was highest (72%) among initiators of denosumab, followed by initiators of alendronate (59%). Persistence with initial treatment was highest after 1 year, decreasing afterwards. We identified several factors associated with higher 2-year persistence for alendronate and denosumab, including older age, female sex, region of residence, employment status, and history of DXA scan procedure. Dementia was associated with higher persistence for alendronate but not denosumab; the opposite was observed for prior OTx. Comorbidities such as COPD, chronic renal impairment, and diabetes were associated with lower persistence for alendronate, but not denosumab. The use of anxiolytics, sedatives, and opioids was associated with lower, whereas the use of statins and antipsychotics was associated with higher persistence of both alendronate and denosumab.

## Limitations

In this population-based study in a setting with universal single-payer health care and complete follow-up, selection bias is assumed to be low. As the use of intravenous zoledronic acid, primarily administered in hospitals, may not be completely captured in the available data sources, it is possible that some patients are misclassified as having no prior use of osteoporosis medications [28]. However, in 2018, the overall use including both hospital and outpatient dispensing of zoledronate among persons 50 years or older accounted for less than 1% of the total use of anti-osteoporosis medication (public access through www.medstat.dk, accessed December 2018). Zoledronic acid is recommended among persons with low-energy hip fractures, those in long-term steroid treatment, or in Paget’s disease patients. Regarding hip fracture patients, very few receive zoledronic acid at the hospital because they are older, more likely to have impaired kidney function and other comorbidities, and because injection should be given 2 weeks after surgery to have the best effect on bone mineral density [29].

Misclassification of the persistence/non-persistence status due to lack of information on the prescribed daily dose and lack of information about the correspondence between the dispensed medicine and the patient’s drug intake, including uncertainty about the start and end of each dispensing is possible. The start and end of each dispensing are estimated based on assumptions that treatment onset corresponds to dispensing date and that the prescribed dose equals the defined daily dose. At least some differences in persistence among the study cohorts may be due to the route of administration, as patients dispensing denosumab are assumed to have received the injection and are therefore by definition persistent for 180 days after each injection. However, sensitivity analyses showed the robustness of study results irrespective of the assumed coverage of a given dispensing and grace periods.

## Comparison with other studies

In the present study, 68% and 59% of patients were persistent with alendronate after 1 and 2 years. These estimates are higher than those presented in a previous Danish study among patients treated during 1996–2006 [10]. In addition, persistence in our study is much higher than persistence reported in other countries. In Sweden, respective numbers are 51% and 35% among patients treated between 2005 and 2009 [13]. In Spain, among patients treated in 2012, persistence was 48% and 29%, respectively [12]. In a literature review and meta-analysis, Karlsson et al. [11] identified 40 studies and calculated pooled 1-year and 2-year persistence with oral bisphosphonates of 45% and 30%.The latest review of Fatoye et al. [14] identified 89 studies within 15 countries and reported persistence to oral bisphosphonates to range from 18 to 75% and from 13 to 72% after 1 and 2 years, respectively.

Persistence for denosumab has been reported only in a few studies. Karlsson et al. [11] reported 1-year and 2-year persistence with denosumab in Sweden during 2010–2012 of 83% and 62%, respectively. Data from Spain showed persistence with denosumab of 66% and 45%, respectively. In addition, among 21,154 women above 40 years treated with denosumab between 2010 and 2014 in Germany, persistence was 56% and 40% after 1 and 2 years [9]. In the UK, based on a cohort of 72,256 women treated during 2010–2015, the persistence to denosumab was 64% and 50% after 1 and 2 years [30]. A small study from the USA and Canada based on 250 women showed persistence of 91% and 97%, respectively [31]. Again, the persistence with denosumab in our study (84% and 72% for 1 and 2 years, respectively) is higher compared to the previous report. The relatively high persistence in Denmark compared to other countries of both alendronate and denosumab could be explained by the differences in reimbursement of treatment rules in different countries, as well as the ability to have a complete follow-up of all treated patients in Denmark. In addition, differences regarding the definition of non-persistence, and treatment of switching and deaths in the analyses could potentially explain these differences.

Both our and previous studies reported higher persistence to denosumab compared to alendronate. This could be explained by the way denosumab is being administered, as an injection every 6 months opposite to weekly treatment with alendronate. In addition, adverse effects are more commonly reported with alendronate compared to denosumab treatment.

Females had higher persistence in our study than males, which is in accordance with some [13, 14] but not all studies [10]. In addition, some studies include only women being unable to study sex differences in persistence [9, 11, 30]. Greater persistence in our study was associated with older ages, which has not been observed in previous studies [10, 13, 14]. In general, older patients in Denmark can have help from general practitioners and home nurses to administrate and control their medication, making medication packages for each day 1 week at a time and delivering the same to their home, increasing the adherence to drugs. Primary non-adherence in general practice in Denmark has been examined by Pottegaard A et al. [32] showing that the overall non-adherence was 9%, and age was found to be the most important predictor of non-adherence.

We observed a difference in persistence with OTx agent by region of residence with capital region in Denmark being the least persistent. This is an unexpected finding as a past Swedish study observed that urban region is a determinant of non-persistence [13]. There is a higher population density and higher patient-per-general practitioner ratio within the capital region which may explain this finding (www. Statbank.dk).

In our study, CCI was not associated with the treatment persistence of any OT agent, which is surprising since the comorbidity burden was previously found to be associated with prematurely terminating OT therapy [10, 13]. However, several individual comorbidities such as COPD, chronic renal impairment, and diabetes were identified as factors associated with lower persistence of alendronate, which is in accordance with previous findings [10, 14]. These conditions were not associated with the persistence of denosumab, which could be explained by the administration form for denosumab; thus, injection is given every 6 months by a general practitioner or home nurse. Dementia was associated with higher persistence with alendronate most likely due to treatment being administrated and controlled by general practitioners or home nurses. On the other hand, we do not have a clear explanation for why dementia was associated with lower persistence of denosumab. It is possible that this finding is related to the severity of dementia and indication for denosumab treatment, of difficulties in remembering the longer interval between treatment administration. However, we did not have information on the severity of dementia in our data.

Concomitant medication such as steroid, anxiolytics and sedatives, and opioids were associated with lower persistence in our study. Two previous studies have reported the same findings for steroid [10, 13] as well as a review by Fatoye et al. [14]. It is likely that patients with chronic anxiety and pain have less surplus to take care of their osteoporosis than patients without these conditions based on the fact that their healthcare utilization is substantially higher compared with the healthcare utilization of individuals without these conditions [33]. This is in accordance with our findings suggesting that a higher number of outpatient and to some extent emergency room visits, where anxiety and pain patients are likely to be treated, are associated with lower persistence.

We observed that prior OT was associated with lower persistence with alendronate which is opposite to previous findings by Hansen et al. [10] based on patients treated from 1996 to 2006. They suggested that physicians should target alendronate to patients who are less likely to discontinue the treatment, for example, patients without pre-existing gastrointestinal problems. It is possible that physicians have applied this strategy in clinical practice, which we now can see the results of among patients treated from 2010 to 2018. We observed that prior OT was associated with higher persistence with denosumab. It is likely that denosumab has been selectively prescribed to patients who had some adverse events with previous treatment and, therefore, are willing to take treatment with less adverse events. This finding is also in the line with findings by Morley et al. [30] and Hadji et al. [9], which showed better persistence and increased preferences and satisfaction with treatment in patients when switched from alendronate to denosumab compared to switching from denosumab to alendronate.

## Conclusion

Persistence with initial osteoporosis treatment was highest within 1 year of initiation, decreasing thereafter. Persistence was highest for denosumab followed by alendronate. We identified several factors associated with treatment persistence, some of which were the same irrespective of the specific OTx agent. The study provided new knowledge about initiators of different OTx agents, as well as factors of association with persistence which could help target subgroups of patients in terms of social and healthcare support.

### Supplementary Information

Below is the link to the electronic supplementary material.

Supplementary file1 (DOCX 94 KB)

## Data Availability

Please see supplementary Table 1 for International Classification of Diseases, Tenth Revision codes, procedure, and NOMESCO surgery codes, and Anatomical Therapeutic Chemical classification system codes used in this study.
