# Supplementary material for: Utilization patterns and factors associated with persistence of new users of anti-osteoporosis treatment in Denmark: a population-based cohort study
Source: Arch Osteoporos. 2023 Jan 11;18(1):19. doi: 10.1007/s11657-023-01210-4 (PMC9834110; doi:10.1007/s11657-023-01210-4)
Supplement: Supplementary file 1 — Supplementary file1 (DOCX 94 KB) [file 11657_2023_1210_MOESM1_ESM.docx]

| **Supplementary Table 1. Baseline Characteristics of New Users of Alendronate, Risedronate, Ibandronate and Denosumab Restricted to patients without a history of Malignancy** | | | | | | | | |
| --- | --- | --- | --- | --- | --- | --- | --- | --- |
|  | **Alendronate** | | **Risedronate** | | **Ibandronate** | | **Denosumab** | |
|  | **N=109,014** | | **N=764** | | **N=5,016** | | **N=13,866** | |
|  | **N** | **%** | **N** | **%** | **N** | **%** | **N** | **%** |
| Sex, n (%) |  |  |  |  |  |  |  |  |
| Female | 86,098 | 79.0 | 667 | 87.3 | 4,461 | 88.9 | 12,443 | 89.7 |
| Male | 22,916 | 21.0 | 97 | 12.7 | 555 | 11.1 | 1,423 | 10.3 |
| Age group, n (%) |  |  |  |  |  |  |  |  |
| 50-<55 | 6,327 | 5.8 | 43 | 5.6 | 242 | 4.8 | 505 | 3.6 |
| 55-<60 | 11,143 | 10.2 | 78 | 10.2 | 543 | 10.8 | 1,063 | 7.7 |
| 60-<65 | 15,165 | 13.9 | 108 | 14.1 | 706 | 14.1 | 1,645 | 11.9 |
| 65-<70 | 19,260 | 17.7 | 154 | 20.2 | 906 | 18.1 | 2,376 | 17.1 |
| 70-<75 | 19,030 | 17.5 | 136 | 17.8 | 945 | 18.8 | 2,487 | 17.9 |
| 75+ | 38,089 | 34.9 | 245 | 32.1 | 1,674 | 33.4 | 5,790 | 41.8 |
| Year of treatment initiation, n (%) |  |  |  |  |  |  |  |  |
| 2010 | 8,220 | 7.5 | 48 | 6.3 | 580 | 11.6 | 428 | 3.1 |
| 2011 | 14,526 | 13.3 | 179 | 23.4 | 846 | 16.9 | 1,940 | 14.0 |
| 2012 | 13,471 | 12.4 | 103 | 13.5 | 546 | 10.9 | 1,936 | 14.0 |
| 2013 | 12,803 | 11.7 | 67 | 8.8 | 543 | 10.8 | 1,708 | 12.3 |
| 2014 | 12,345 | 11.3 | 97 | 12.7 | 557 | 11.1 | 1,783 | 12.9 |
| 2015 | 12,060 | 11.1 | 35 | 4.6 | 521 | 10.4 | 1,770 | 12.8 |
| 2016 | 12,061 | 11.1 | 65 | 8.5 | 480 | 9.6 | 1,489 | 10.7 |
| 2017 | 11,790 | 10.8 | 78 | 10.2 | 454 | 9.1 | 1,430 | 10.3 |
| 2018 | 11,738 | 10.8 | 92 | 12.0 | 489 | 9.7 | 1,382 | 10.0 |
| Region of residence, n (%) |  |  |  |  |  |  |  |  |
| Capital | 26,561 | 24.4 | 242 | 31.7 | 1,155 | 23.0 | 3,347 | 24.1 |
| Zealand | 15,383 | 14.1 | 79 | 10.3 | 963 | 19.2 | 1,616 | 11.7 |
| Southern Denmark | 25,615 | 23.5 | 156 | 20.4 | 885 | 17.6 | 2,446 | 17.6 |
| Central Jutland | 27,370 | 25.1 | 187 | 24.5 | 1,339 | 26.7 | 4,370 | 31.5 |
| Northern Jutland | 14,085 | 12.9 | 100 | 13.1 | 674 | 13.4 | 2,087 | 15.1 |
| Annual household income, n (%) |  |  |  |  |  |  |  |  |
| <200.000 kr. | 18,541 | 17.0 | 131 | 17.1 | 820 | 16.3 | 2,492 | 18.0 |
| 200.000-300.000 kr. | 30,894 | 28.3 | 198 | 25.9 | 1,433 | 28.6 | 4,224 | 30.5 |
| 300.000-400.000 kr. | 18,906 | 17.3 | 127 | 16.6 | 889 | 17.7 | 2,408 | 17.4 |
| ≥400.000 kr. | 40,563 | 37.2 | 308 | 40.3 | 1,874 | 37.4 | 4,742 | 34.2 |
| Missing | 110 | 0.1 | ↑ |  | ↑ |  | ↑ |  |
| Educational level, n (%) |  |  |  |  |  |  |  |  |
| Primary school | 45,114 | 41.4 | 281 | 36.8 | 2,058 | 41.0 | 5,803 | 41.9 |
| Secondary school | 41,399 | 38.0 | 295 | 38.6 | 1,938 | 38.6 | 5,043 | 36.4 |
| Higher | 19,599 | 18.0 | 171 | 22.4 | 884 | 17.6 | 2,664 | 19.2 |
| Missing | 2,902 | 2.7 | 17 | 2.2 | 136 | 2.7 | 356 | 2.6 |
| Employment status, n (%) |  |  |  |  |  |  |  |  |
| Director/Chief Executive | 9,021 | 8.3 | 72 | 9.4 | 363 | 7.2 | 840 | 6.1 |
| Employer/Self employed | 2,884 | 2.6 | 21 | 2.7 | 128 | 2.6 | 295 | 2.1 |
| Skilled worker | 7,877 | 7.2 | 41 | 5.4 | 307 | 6.1 | 593 | 4.3 |
| Unskilled worker | 1,827 | 1.7 | 11 | 1.4 | 68 | 1.4 | 115 | 0.8 |
| Early retirement/pension | 81,429 | 74.7 | 585 | 76.6 | 3,865 | 77.1 | 11,470 | 82.7 |
| Unemployed, benefits/public support | 2,665 | 2.4 | 11 | 1.4 | 107 | 2.1 | 193 | 1.4 |
| Other | 3,311 | 3.0 | 23 | 3.0 | 178 | 3.5 | 360 | 2.6 |
| Missing | 0 | 0.0 | 0 | 0.0 | 0 | 0.0 | 0 | 0.0 |
| **Comorbidities** |  |  |  |  |  |  |  |  |
| Charlson comorbidity index as a measure of the overall hospital comorbidity (CCI),  n (%) |  |  |  |  |  |  |  |  |
| Low | 76,166 | 69.9 | 524 | 68.6 | 3,627 | 72.3 | 9,273 | 66.9 |
| Medium | 28,250 | 25.9 | 202 | 26.4 | 1,225 | 24.4 | 3,801 | 27.4 |
| High | 4,598 | 4.2 | 38 | 5.0 | 164 | 3.3 | 792 | 5.7 |
| Individual comorbidities, n (%) |  |  |  |  |  |  |  |  |
| Chronic obstructive pulmonary disease | 13,109 | 12.0 | 86 | 11.3 | 603 | 12.0 | 1,852 | 13.4 |
| Chronic renal impairment | 1,342 | 1.2 | 11 | 1.4 | 26 | 0.5 | 392 | 2.8 |
| Diabetes | 9,843 | 9.0 | 59 | 7.7 | 335 | 6.7 | 1,075 | 7.8 |
| Dementia | 2,051 | 1.9 | 7 | 0.9 | 75 | 1.5 | 336 | 2.4 |
| **Osteoporosis-related characteristics** |  |  |  |  |  |  |  |  |
| Hospital diagnosis of osteoporosis (OP), n (%) | 41,339 | 37.9 | 398 | 52.1 | 2,154 | 42.9 | 10,110 | 72.9 |
| Months from hospital diagnosis of osteoporosis to index date (among patients with  hospital dx of OP) |  |  |  |  |  |  |  |  |
| 0-1 | 10,980 | 10.1 | 23 | 3.0 | 87 | 1.7 | 341 | 2.5 |
| >1-6 | 18,809 | 17.3 | 86 | 11.3 | 451 | 9.0 | 1,719 | 12.4 |
| >6-12 | 2,614 | 2.4 | 55 | 7.2 | 288 | 5.7 | 1,264 | 9.1 |
| >12 | 8,936 | 8.2 | 234 | 30.6 | 1,328 | 26.5 | 6,786 | 48.9 |
| Missing | 67,675 | 62.1 | 366 | 47.9 | 2,862 | 57.1 | 3,756 | 27.1 |
| History of hip fracture, n (%) | 9,986 | 9.2 | 42 | 5.5 | 324 | 6.5 | 1,422 | 10.3 |
| History of vertebral fracture, n (%) | 3,488 | 3.2 | 19 | 2.5 | 154 | 3.1 | 741 | 5.3 |
| History of forearm fracture, n (%) | 16,214 | 14.9 | 131 | 17.1 | 780 | 15.6 | 2,646 | 19.1 |
| History of humerus fracture, n (%) | 6,744 | 6.2 | 45 | 5.9 | 333 | 6.6 | 1,128 | 8.1 |
| Months from diagnosis of last major osteoporotic fracture to index date (among  patients with OP fracture) |  |  |  |  |  |  |  |  |
| 0-1 | 1,537 | 1.4 | 17 | 2.2 | 10 | 0.2 | 39 | 0.3 |
| >1-6 | 5,079 | 4.7 | ↑ |  | 87 | 1.7 | 251 | 1.8 |
| >6-12 | 2,515 | 2.3 | 13 | 1.7 | 118 | 2.4 | 305 | 2.2 |
| >12 | 21,338 | 19.6 | 167 | 21.9 | 1,125 | 22.4 | 4,100 | 29.6 |
| Missing | 78,545 | 72.1 | 567 | 74.2 | 3,676 | 73.3 | 9,171 | 66.1 |
| DXA scan procedure, n (%) | 85,037 | 78.0 | 667 | 87.3 | 4,416 | 88.0 | 12,865 | 92.8 |
| Months from last DXA procedure to index date (among patients with DXA) |  |  |  |  |  |  |  |  |
| 0-1 | 28,085 | 25.8 | 32 | 4.2 | 319 | 6.4 | 225 | 1.6 |
| >1-6 | 25,990 | 23.8 | 134 | 17.5 | 921 | 18.4 | 1,647 | 11.9 |
| >6-12 | 2,805 | 2.6 | 75 | 9.8 | 423 | 8.4 | 1,133 | 8.2 |
| >12 | 28,157 | 25.8 | 426 | 55.8 | 2,753 | 54.9 | 9,860 | 71.1 |
| Missing | 23,977 | 22.0 | 97 | 12.7 | 600 | 12.0 | 1,001 | 7.2 |
| **Prior Osteoporosis treatment (OT)x, n (%)** |  |  |  |  |  |  |  |  |
| All OTx | 5,277 | 4.8 | 646 | 84.6 | 4,074 | 81.2 | 12,021 | 86.7 |
| Raloxifene | 747 | 0.7 | 13 | 1.7 | 110 | 2.2 | 647 | 4.7 |
| Teriparatide | 1,031 | 0.9 | 20 | 2.6 | 79 | 1.6 | 950 | 6.9 |
| Parathyroid hormone | 103 | 0.1 | 6 | 0.8 | 9 | 0.2 | 152 | 1.1 |
| Etidronate | 2,306 | 2.1 | 54 | 7.1 | 273 | 5.4 | 1,272 | 9.2 |
| Alendronate | 0 | 0.0 | 634 | 83.0 | 3,973 | 79.2 | 11,227 | 81.0 |
| Ibandronate | 738 | 0.7 | 41 | 5.4 | 0 | 0.0 | 1,603 | 11.6 |
| Risedronate | 287 | 0.3 | 0 | 0.0 | 80 | 1.6 | 331 | 2.4 |
| Zoledronic acid | <5 |  | <5 |  | <5 |  | 32 | 0.2 |
| Strontium ranelate | 498 | 0.5 | 22 | 2.9 | 168 | 3.3 | 911 | 6.6 |
| Denosumab | 133 | 0.1 | 24 | 3.1 | 73 | 1.5 | 0 | 0.0 |
| Months from last dispensing of prior OTx to index date (among patients with prior  use) |  |  |  |  |  |  |  |  |
| 0-1 | 40 | 0.0 | 37 | 4.8 | 205 | 4.1 | 108 | 0.8 |
| >1-6 | 160 | 0.1 | 180 | 23.6 | 998 | 19.9 | 1,476 | 10.6 |
| >6-12 | 108 | 0.1 | 73 | 9.6 | 554 | 11.0 | 1,062 | 7.7 |
| >12 | 4,969 | 4.6 | 356 | 46.6 | 2,317 | 46.2 | 9,375 | 67.6 |
| Missing | 103,737 | 95.2 | 118 | 15.4 | 942 | 18.8 | 1,845 | 13.3 |
| **Concomitant medication, n (%)** |  |  |  |  |  |  |  |  |
| Oral corticosteroids | 28,031 | 25.7 | 165 | 21.6 | 1,049 | 20.9 | 2,276 | 16.4 |
| Anticoagulants | 8,869 | 8.1 | 45 | 5.9 | 313 | 6.2 | 1,174 | 8.5 |
| Antidiabetics | 7,658 | 7.0 | 47 | 6.2 | 251 | 5.0 | 760 | 5.5 |
| Antithrombotics | 28,117 | 25.8 | 181 | 23.7 | 1,210 | 24.1 | 3,776 | 27.2 |
| Hormone replacement theory | 16,663 | 15.3 | 157 | 20.5 | 1,016 | 20.3 | 2,844 | 20.5 |
| Hormone deprivation theory | 7 | 0.0 | 0 | 0.0 | 0 | 0.0 | <5 |  |
| Anxiolytics and sedatives | 21,615 | 19.8 | 162 | 21.2 | 1,183 | 23.6 | 3,399 | 24.5 |
| Antipsychotics | 3,911 | 3.6 | 22 | 2.9 | 165 | 3.3 | 507 | 3.7 |
| Antidepressants | 19,003 | 17.4 | 107 | 14.0 | 934 | 18.6 | 2,812 | 20.3 |
| Statins | 32,743 | 30.0 | 207 | 27.1 | 1,384 | 27.6 | 4,065 | 29.3 |
| Non-steroid anti-inflammatory drugs | 34,918 | 32.0 | 209 | 27.4 | 1,521 | 30.3 | 3,457 | 24.9 |
| Antihypersentive drugs | 59,505 | 54.6 | 385 | 50.4 | 2,617 | 52.2 | 7,717 | 55.7 |
| Drugs for treatment of chronic obstructive pulmonary disease | 31,349 | 28.8 | 237 | 31.0 | 1,588 | 31.7 | 4,283 | 30.9 |
| Opioids | 38,830 | 35.6 | 198 | 25.9 | 1,652 | 32.9 | 5,079 | 36.6 |
| Anti-thyroid drugs | 2,057 | 1.9 | 11 | 1.4 | 114 | 2.3 | 275 | 2.0 |
| **Health care utilization in the year before index date, n (%)** |  |  |  |  |  |  |  |  |
| Number of hospitalizations |  |  |  |  |  |  |  |  |
| 0 | 73,797 | 67.7 | 599 | 78.4 | 3,882 | 77.4 | 9,688 | 69.9 |
| 1 | 22,254 | 20.4 | 124 | 16.2 | 775 | 15.5 | 2,406 | 17.4 |
| >1 | 12,963 | 11.9 | 41 | 5.4 | 359 | 7.2 | 1,772 | 12.8 |
| Number of outpatient visits |  |  |  |  |  |  |  |  |
| 0 | 33,127 | 30.4 | 225 | 29.5 | 1,929 | 38.5 | 2,873 | 20.7 |
| 1 | 23,067 | 21.2 | 165 | 21.6 | 978 | 19.5 | 2,876 | 20.7 |
| >1 | 52,820 | 48.5 | 374 | 49.0 | 2,109 | 42.0 | 8,117 | 58.5 |
| Number of emergency room visits |  |  |  |  |  |  |  |  |
| 0 | 83,686 | 76.8 | 633 | 82.9 | 4,220 | 84.1 | 11,081 | 79.9 |
| 1 | 19,283 | 17.7 | 103 | 13.5 | 639 | 12.7 | 2,074 | 15.0 |
| >1 | 6,045 | 5.5 | 28 | 3.7 | 157 | 3.1 | 711 | 5.1 |

| Supplementary Table 2. Factors associated with persistence after 2 Years By Treatment Cohort Among Patients Without Malignancy at Baseline Initiating Treatment in 2010-2016 | | | | | | | | | | | | | | | | |
| --- | --- | --- | --- | --- | --- | --- | --- | --- | --- | --- | --- | --- | --- | --- | --- | --- |
|  |  |  |  |  |  |  |  |  |  |  |  |  |  |  |  |  |
|  | Alendronate | | | | Risedronate | | | | Ibandronate | | | | Denosumab | | | |
|  | N | n | Crude OR (95% CI) | Multivariable OR (95% CI) | N | n | Crude OR (95% CI) | Multivariable OR (95% CI) | N | n | Crude OR (95% CI) | Multivariable OR (95% CI) | N | n | Crude OR (95% CI) | Multivariable OR (95% CI) |
| Sex |  |  |  |  |  |  |  |  |  |  |  |  |  |  |  |  |
| Female | 67,697 | 40,720 | 1.17 (1.13-1.21) | 1.13 (1.09-1.18) | 523 | 152 | 1.12 (0.64-1.96) | 1.38 (0.71-2.66) | 3,617 | 1,585 | 0.96 (0.79-1.17) | 1.00 (0.81-1.24) | 9,911 | 7,220 | 1.21 (1.06-1.39) | 1.12 (0.97-1.29) |
| Male | 17,789 | 10,029 | Ref | Ref | 71 | 19 | Ref | Ref | 456 | 204 | Ref | Ref | 1,143 | 787 | Ref | Ref |
| Age group |  |  |  |  |  |  |  |  |  |  |  |  |  |  |  |  |
| 50-<55 | 4,924 | 2,590 | Ref | Ref | 33 | 11 | Ref | Ref | 192 | 67 | Ref | Ref | 412 | 287 | Ref | Ref |
| 55-<60 | 8,654 | 4,837 | 1.14 (1.06-1.23) | 1.14 (1.06-1.22) | 60 | 12 | 0.50 (0.19-1.31) | 0.52 (0.18-1.57) | 446 | 176 | 1.22 (0.86-1.73) | 1.23 (0.85-1.76) | 879 | 620 | 1.04 (0.81-1.35) | 1.04 (0.80-1.35) |
| 60-<65 | 11,744 | 7,011 | 1.33 (1.25-1.43) | 1.36 (1.27-1.46) | 85 | 24 | 0.79 (0.33-1.87) | 0.99 (0.36-2.72) | 559 | 255 | 1.56 (1.11-2.20) | 1.52 (1.07-2.18) | 1,337 | 976 | 1.18 (0.92-1.50) | 1.19 (0.93-1.53) |
| 65-<70 | 15,442 | 9,392 | 1.40 (1.31-1.49) | 1.49 (1.39-1.60) | 125 | 40 | 0.94 (0.42-2.13) | 1.06 (0.39-2.91) | 773 | 352 | 1.56 (1.12-2.17) | 1.62 (1.14-2.32) | 1,929 | 1,468 | 1.39 (1.10-1.75) | 1.45 (1.13-1.86) |
| 70-<75 | 14,481 | 8,736 | 1.37 (1.28-1.46) | 1.54 (1.43-1.66) | 107 | 29 | 0.74 (0.32-1.72) | 0.93 (0.32-2.67) | 733 | 333 | 1.55 (1.12-2.16) | 1.62 (1.13-2.34) | 1,944 | 1,466 | 1.34 (1.06-1.69) | 1.49 (1.16-1.92) |
| 75+ | 30,241 | 18,183 | 1.36 (1.28-1.44) | 1.51 (1.41-1.62) | 184 | 55 | 0.85 (0.39-1.88) | 1.13 (0.39-3.28) | 1,370 | 606 | 1.48 (1.08-2.03) | 1.71 (1.19-2.46) | 4,553 | 3,190 | 1.02 (0.82-1.27) | 1.26 (0.99-1.61) |
| Year of treatment initiation | | | | | | | | | | | | | | | | |
| 2010 | 8,220 | 5,044 | Ref | Ref | 48 | 11 | Ref | Ref | 580 | 265 | Ref | Ref | 428 | 303 | Ref | Ref |
| 2011 | 14,526 | 8,704 | 0.94 (0.89-1.00) | 0.95 (0.89-1.00) | 179 | 62 | 1.78 (0.85-3.74) | 1.79 (0.77-4.18) | 846 | 388 | 1.01 (0.81-1.24) | 0.97 (0.77-1.21) | 1,940 | 1,435 | 1.17 (0.93-1.48) | 1.20 (0.95-1.53) |
| 2012 | 13,471 | 7,891 | 0.89 (0.84-0.94) | 0.88 (0.83-0.93) | 103 | 28 | 1.26 (0.56-2.80) | 1.26 (0.52-3.06) | 546 | 233 | 0.88 (0.70-1.12) | 0.88 (0.69-1.13) | 1,936 | 1,348 | 0.95 (0.75-1.19) | 0.94 (0.74-1.19) |
| 2013 | 12,803 | 7,552 | 0.91 (0.86-0.96) | 0.89 (0.84-0.94) | 67 | 14 | 0.89 (0.36-2.17) | 0.97 (0.36-2.65) | 543 | 224 | 0.83 (0.66-1.06) | 0.85 (0.66-1.09) | 1,708 | 1,240 | 1.09 (0.87-1.38) | 1.06 (0.83-1.35) |
| 2014 | 12,345 | 7,338 | 0.92 (0.87-0.98) | 0.91 (0.85-0.96) | 97 | 22 | 0.99 (0.43-2.25) | 0.88 (0.35-2.26) | 557 | 245 | 0.93 (0.74-1.18) | 0.97 (0.76-1.24) | 1,783 | 1,297 | 1.10 (0.87-1.39) | 1.07 (0.84-1.37) |
| 2015 | 12,060 | 7,178 | 0.93 (0.87-0.98) | 0.90 (0.85-0.96) | 35 | 10 | 1.35 (0.50-3.64) | 1.24 (0.38-4.03) | 521 | 221 | 0.88 (0.69-1.11) | 0.85 (0.66-1.09) | 1,770 | 1,290 | 1.11 (0.88-1.40) | 1.10 (0.86-1.40) |
| 2016 | 12,061 | 7,042 | 0.88 (0.83-0.94) | 0.86 (0.81-0.91) | 65 | 24 | 1.97 (0.85-4.56) | 1.56 (0.60-4.06) | 480 | 213 | 0.95 (0.74-1.21) | 0.92 (0.71-1.19) | 1,489 | 1,094 | 1.14 (0.90-1.45) | 1.11 (0.87-1.43) |
| Region of residence | | | |  |  |  |  |  |  |  |  |  |  |  |  |  |
| Capital | 21,040 | 11,660 | Ref | Ref | 201 | 52 | Ref | Ref | 980 | 417 | Ref | Ref | 2,814 | 1,901 | Ref | Ref |
| Zealand | 12,082 | 7,021 | 1.12 (1.07-1.17) | 1.13 (1.08-1.18) | 62 | 15 | 0.91 (0.47-1.77) | 0.77 (0.36-1.67) | 775 | 348 | 1.10 (0.91-1.33) | 1.09 (0.89-1.33) | 1,244 | 895 | 1.23 (1.06-1.43) | 1.21 (1.04-1.42) |
| Southern  Denmark | 20,244 | 12,166 | 1.21 (1.17-1.26) | 1.19 (1.14-1.24) | 126 | 41 | 1.38 (0.85-2.25) | 1.27 (0.70-2.31) | 717 | 330 | 1.15 (0.95-1.40) | 1.26 (1.01-1.57) | 1,908 | 1,359 | 1.19 (1.05-1.35) | 1.18 (1.03-1.35) |
| Central Jutland | 21,481 | 13,197 | 1.28 (1.23-1.33) | 1.27 (1.22-1.32) | 132 | 37 | 1.12 (0.68-1.83) | 1.23 (0.67-2.25) | 1,084 | 473 | 1.05 (0.88-1.24) | 1.06 (0.88-1.28) | 3,608 | 2,702 | 1.43 (1.28-1.60) | 1.39 (1.24-1.56) |
| Northern Jutland | 10,639 | 6,705 | 1.37 (1.31-1.44) | 1.33 (1.27-1.40) | 73 | 26 | 1.59 (0.89-2.81) | 1.29 (0.63-2.63) | 517 | 221 | 1.01 (0.81-1.25) | 1.06 (0.84-1.34) | 1,480 | 1,150 | 1.67 (1.45-1.94) | 1.57 (1.35-1.83) |
| Annual household income | | | |  |  |  |  |  |  |  |  |  |  |  |  |  |
| <200.000 kr. | 16,321 | 9,740 | Ref | Ref | 112 | 32 | Ref | Ref | 742 | 297 | Ref | Ref | 2,166 | 1,521 | Ref | Ref |
| 200.000-300.000  kr. | 24,472 | 14,343 | 0.96 (0.92-1.00) | 0.98 (0.94-1.03) | 151 | 48 | 1.17 (0.68-1.99) | 1.18 (0.64-2.20) | 1,173 | 531 | 1.24 (1.03-1.49) | 1.33 (1.09-1.62) | 3,363 | 2,372 | 1.02 (0.90-1.14) | 0.97 (0.86-1.10) |
| 300.000-400.000  kr. | 14,381 | 8,481 | 0.97 (0.93-1.02) | 1.04 (0.99-1.09) | 97 | 25 | 0.87 (0.47-1.60) | 1.20 (0.58-2.50) | 708 | 319 | 1.23 (1.00-1.51) | 1.34 (1.06-1.68) | 1,867 | 1,385 | 1.22 (1.06-1.40) | 1.12 (0.96-1.30) |
| ≥400.000 kr. | 30,222 | 18,140 | 1.01 (0.98-1.05) | 1.15 (1.09-1.21) | 234 | 66 | 0.99 (0.60-1.63) | 1.27 (0.64-2.53) | 1,450 | 642 | 1.19 (0.99-1.42) | 1.35 (1.08-1.69) | 3,658 | 2,729 | 1.25 (1.11-1.40) | 1.13 (0.98-1.31) |
| Educational level |  |  |  |  |  |  |  |  |  |  |  |  |  |  |  |  |
| Primary school | 36,693 | 22,106 | Ref | Ref | 231 | 62 | Ref | Ref | 1,718 | 751 | Ref | Ref | 4,702 | 3,370 | Ref | Ref |
| Secondary  school | 31,676 | 18,699 | 0.95 (0.92-0.98) | 0.97 (0.93-1.00) | 221 | 66 | 1.16 (0.77-1.75) | 1.28 (0.80-2.06) | 1,534 | 696 | 1.07 (0.93-1.23) | 1.06 (0.91-1.23) | 3,966 | 2,908 | 1.09 (0.99-1.19) | 1.05 (0.95-1.16) |
| Higher | 14,611 | 8,541 | 0.93 (0.89-0.97) | 0.91 (0.87-0.95) | 126 | 37 | 1.13 (0.70-1.83) | 1.32 (0.73-2.40) | 700 | 297 | 0.95 (0.79-1.13) | 0.92 (0.76-1.13) | 2,087 | 1,515 | 1.05 (0.93-1.17) | 0.98 (0.86-1.11) |
| Employment  status | | |  |  |  |  |  |  |  |  |  |  |  |  |  |  |
| Employed | 16,278 | 9,596 | 0.98 (0.95-1.01) | 1.09 (1.04-1.14) | 110 | 31 | 0.96 (0.61-1.53) | 1.10 (0.56-2.14) | 669 | 282 | 0.92 (0.78-1.08) | 1.03 (0.84-1.28) | 1,438 | 1,071 | 1.13 (0.99-1.28) | 1.10 (0.94-1.28) |
| Unemployed | 69,208 | 41,153 | Ref | Ref | 484 | 140 | Ref | Ref | 3,404 | 1,507 | Ref | Ref | 9,616 | 6,936 | Ref | Ref |
| Comorbidities |  |  |  |  |  |  |  |  |  |  |  |  |  |  |  |  |
| Charlson comorbidity index (CCI) | | | |  |  |  |  |  |  |  |  |  |  |  |  |  |
| Low | 59,621 | 36,119 | Ref | Ref | 398 | 110 | Ref | Ref | 2,925 | 1,318 | Ref | Ref | 7,387 | 5,508 | Ref | Ref |
| Medium | 22,253 | 12,500 | 0.83 (0.81-0.86) | 0.94 (0.90-0.97) | 167 | 46 | 1.00 (0.66-1.49) | 0.98 (0.56-1.72) | 1,012 | 427 | 0.89 (0.77-1.03) | 0.90 (0.76-1.08) | 3,031 | 2,066 | 0.73 (0.67-0.80) | 0.85 (0.76-0.95) |
| High | 3,612 | 2,130 | 0.94 (0.87-1.00) | 1.02 (0.94-1.10) | 29 | 15 | 2.81 (1.31-6.00) | 3.20 (1.13-9.10) | 136 | 44 | 0.58 (0.40-0.84) | 0.61 (0.41-0.92) | 636 | 433 | 0.73 (0.61-0.87) | 1.04 (0.84-1.28) |
| Individual comorbidities | | | |  |  |  |  |  |  |  |  |  |  |  |  |  |
| Chronic obstructive pulmonary disease, including drugs for COPD treatment | | | | | | | | | | | | | | | | |
| No | 59,549 | 36,123 | Ref | Ref | 395 | 113 | Ref | Ref | 2,718 | 1,199 | Ref | Ref | 7,423 | 5,458 | Ref | Ref |
| Yes | 25,937 | 14,626 | 0.84 (0.81-0.86) | 0.91 (0.88-0.94) | 199 | 58 | 1.03 (0.71-1.49) | 0.91 (0.56-1.49) | 1,355 | 590 | 0.98 (0.86-1.11) | 1.07 (0.92-1.24) | 3,631 | 2,549 | 0.85 (0.78-0.93) | 0.98 (0.89-1.08) |
| Chronic renal impairment | | | |  |  |  |  |  |  |  |  |  |  |  |  |  |
| No | 84,449 | 50,191 | Ref | Ref | N/A | N/A | Ref | Ref | 4,051 | 1,779 | Ref | Ref | 10,742 | 7,790 | Ref | Ref |
| Yes | 1,037 | 558 | 0.80 (0.70-0.90) | 0.83 (0.73-0.94) | N/A | N/A | 0.82 (0.16-4.12) | N/A | 22 | 10 | 1.06 (0.46-2.47) | 1.16 (0.47-2.85) | 312 | 217 | 0.87 (0.68-1.11) | 1.06 (0.82-1.38) |
| Diabetes, including antidiabetic medication | | | | |  |  |  |  |  |  |  |  |  |  |  |  |
| No | 77,898 | 46,383 | Ref | Ref | 547 | 156 | Ref | Ref | 3,794 | 1,677 | Ref | Ref | 10,234 | 7,435 | Ref | Ref |
| Yes | 7,588 | 4,366 | 0.92 (0.88-0.97) | 0.92 (0.88-0.97) | 47 | 15 | 1.17 (0.62-2.23) | 0.54 (0.23-1.29) | 279 | 112 | 0.85 (0.66-1.08) | 0.87 (0.66-1.14) | 820 | 572 | 0.87 (0.74-1.01) | 0.98 (0.83-1.16) |
| Dementia |  |  |  |  |  |  |  |  |  |  |  |  |  |  |  |  |
| No | 83,801 | 49,555 | Ref | Ref | N/A | N/A | Ref | Ref | 4,009 | 1,762 | Ref | Ref | 10,779 | 7,841 | Ref | Ref |
| Yes | 1,685 | 1,194 | 1.68 (1.51-1.87) | 1.65 (1.47-1.85) | N/A | N/A | 6.34 (1.22-33.00) | N/A | 64 | 27 | 0.93 (0.56-1.53) | 1.14 (0.65-1.99) | 275 | 166 | 0.57 (0.45-0.73) | 0.75 (0.57-0.98) |
| Osteoporosis (OP) - related characteristics | | | | |  |  |  |  |  |  |  |  |  |  |  |  |
| Hospital diagnosis of osteoporosis | | |  |  |  |  |  |  |  |  |  |  |  |  |  |  |
| No | 52,595 | 30,492 | Ref | Ref | 291 | 90 | Ref | Ref | 2,306 | 1,030 | Ref | Ref | 2,915 | 2,146 | Ref | Ref |
| Yes | 32,891 | 20,257 | 1.16 (1.13-1.20) | 1.10 (1.07-1.14) | 303 | 81 | 0.81 (0.57-1.16) | 1.08 (0.67-1.77) | 1,767 | 759 | 0.93 (0.82-1.06) | 0.94 (0.81-1.10) | 8,139 | 5,861 | 0.92 (0.84-1.01) | 0.92 (0.82-1.03) |
| History of hip fracture |  |  |  |  |  |  |  |  |  |  |  |  |  |  |  |  |
| No | 77,657 | 45,797 | Ref | Ref | 559 | 157 | Ref | Ref | 3,805 | 1,664 | Ref | Ref | 9,943 | 7,233 | Ref | Ref |
| Yes | 7,829 | 4,952 | 1.20 (1.14-1.26) | 1.05 (0.99-1.11) | 35 | 14 | 1.71 (0.85-3.44) | 2.11 (0.90-4.96) | 268 | 125 | 1.12 (0.88-1.44) | 1.09 (0.83-1.43) | 1,111 | 774 | 0.86 (0.75-0.99) | 0.99 (0.86-1.15) |
| History of vertebral fracture | | |  |  |  |  |  |  |  |  |  |  |  |  |  |  |
| No | 82,879 | 49,224 | Ref | Ref | 582 | 166 | Ref | Ref | 3,955 | 1,735 | Ref | Ref | 10,485 | 7,591 | Ref | Ref |
| Yes | 2,607 | 1,525 | 0.96 (0.89-1.04) | 0.96 (0.89-1.05) | 12 | 5 | 1.79 (0.56-5.72) | 2.91 (0.78-10.93) | 118 | 54 | 1.08 (0.75-1.56) | 1.12 (0.76-1.65) | 569 | 416 | 1.04 (0.86-1.25) | 1.18 (0.96-1.44) |
| History of forearm  fracture |  |  |  |  |  |  |  |  |  |  |  |  |  |  |  |  |
| No | 73,082 | 43,119 | Ref | Ref | 498 | 137 | Ref | Ref | 3,456 | 1,508 | Ref | Ref | 8,938 | 6,481 | Ref | Ref |
| Yes | 12,404 | 7,630 | 1.11 (1.07-1.15) | 1.06 (1.02-1.11) | 96 | 34 | 1.45 (0.91-2.29) | 1.51 (0.88-2.61) | 617 | 281 | 1.08 (0.91-1.28) | 1.10 (0.91-1.32) | 2,116 | 1,526 | 0.98 (0.88-1.09) | 1.03 (0.92-1.15) |
| History of  humerus fracture |  |  |  |  |  |  |  |  |  |  |  |  |  |  |  |  |
| No | 80,348 | 47,638 | Ref | Ref | 563 | 157 | Ref | Ref | 3,798 | 1,662 | Ref | Ref | 10,153 | 7,390 | Ref | Ref |
| Yes | 5,138 | 3,111 | 1.05 (0.99-1.12) | 1.02 (0.96-1.09) | 31 | 14 | 2.13 (1.03-4.42) | 3.16 (1.26-7.89) | 275 | 127 | 1.10 (0.86-1.41) | 1.06 (0.82-1.37) | 901 | 617 | 0.81 (0.70-0.94) | 0.89 (0.76-1.04) |
| DXA scan  procedure |  |  |  |  |  |  |  |  |  |  |  |  |  |  |  |  |
| No | 19,407 | 11,505 | Ref | Ref | 81 | 24 | Ref | Ref | 529 | 237 | Ref | Ref | 854 | 600 | Ref | Ref |
| Yes | 66,079 | 39,244 | 1.00 (0.97-1.04) | 1.07 (1.03-1.11) | 513 | 147 | 0.95 (0.57-1.59) | 1.22 (0.63-2.38) | 3,544 | 1,552 | 0.96 (0.80-1.15) | 1.04 (0.85-1.28) | 10,200 | 7,407 | 1.12 (0.96-1.31) | 1.14 (0.96-1.36) |
| Prior OTx |  |  |  |  |  |  |  |  |  |  |  |  |  |  |  |  |
| No | 80,810 | 47,992 | Ref | Ref | 107 | 39 | Ref | Ref | 785 | 416 | Ref | Ref | 1,511 | 1,013 | Ref | Ref |
| Yes | 4,676 | 2,757 | 0.98 (0.93-1.04) | 0.90 (0.85-0.96) | 487 | 132 | 0.65 (0.42-1.01) | 0.55 (0.31-0.98) | 3,288 | 1,373 | 0.64 (0.54-0.74) | 0.62 (0.53-0.74) | 9,543 | 6,994 | 1.35 (1.20-1.52) | 1.22 (1.07-1.38) |
| Concomitant medication | | | | |  |  |  |  |  |  |  |  |  |  |  |  |
| Oral  corticosteroids |  |  |  |  |  |  |  |  |  |  |  |  |  |  |  |  |
| No | 63,476 | 38,839 | Ref | Ref | 467 | 126 | Ref | Ref | 3,207 | 1,430 | Ref | Ref | 9,209 | 6,731 | Ref | Ref |
| Yes | 22,010 | 11,910 | 0.75 (0.73-0.77) | 0.80 (0.77-0.82) | 127 | 45 | 1.49 (0.98-2.25) | 1.70 (1.00-2.88) | 866 | 359 | 0.88 (0.76-1.02) | 0.90 (0.75-1.07) | 1,845 | 1,276 | 0.83 (0.74-0.92) | 0.91 (0.80-1.02) |
| Anticoagulants |  |  |  |  |  |  |  |  |  |  |  |  |  |  |  |  |
| No | 78,866 | 46,757 | Ref | Ref | 562 | 161 | Ref | Ref | 3,830 | 1,688 | Ref | Ref | 10,187 | 7,420 | Ref | Ref |
| Yes | 6,620 | 3,992 | 1.04 (0.99-1.10) | 1.03 (0.97-1.09) | 32 | 10 | 1.13 (0.52-2.44) | 1.12 (0.45-2.82) | 243 | 101 | 0.90 (0.69-1.17) | 0.85 (0.64-1.14) | 867 | 587 | 0.78 (0.67-0.91) | 0.87 (0.74-1.03) |
| Antithrombotics |  |  |  |  |  |  |  |  |  |  |  |  |  |  |  |  |
| No | 62,580 | 37,013 | Ref | Ref | 446 | 121 | Ref | Ref | 3,030 | 1,344 | Ref | Ref | 7,953 | 5,819 | Ref | Ref |
| Yes | 22,906 | 13,736 | 1.03 (1.00-1.07) | 0.99 (0.95-1.03) | 148 | 50 | 1.37 (0.92-2.04) | 1.24 (0.71-2.19) | 1,043 | 445 | 0.93 (0.81-1.08) | 0.83 (0.70-0.99) | 3,101 | 2,188 | 0.88 (0.80-0.96) | 0.92 (0.83-1.03) |
| Hormone  replacement  theory |  |  |  |  |  |  |  |  |  |  |  |  |  |  |  |  |
| No | 72,606 | 43,006 | Ref | Ref | 476 | 140 | Ref | Ref | 3,285 | 1,440 | Ref | Ref | 8,851 | 6,373 | Ref | Ref |
| Yes | 12,880 | 7,743 | 1.04 (1.00-1.08) | 1.02 (0.98-1.07) | 118 | 31 | 0.86 (0.54-1.35) | 1.03 (0.61-1.74) | 788 | 349 | 1.02 (0.87-1.19) | 1.03 (0.88-1.22) | 2,203 | 1,634 | 1.12 (1.00-1.24) | 1.08 (0.96-1.20) |
| Anxiolytics and  sedatives |  |  |  |  |  |  |  |  |  |  |  |  |  |  |  |  |
| No | 67,664 | 40,939 | Ref | Ref | 457 | 139 | Ref | Ref | 3,062 | 1,372 | Ref | Ref | 8,209 | 6,055 | Ref | Ref |
| Yes | 17,822 | 9,810 | 0.80 (0.77-0.83) | 0.80 (0.78-0.83) | 137 | 32 | 0.70 (0.45-1.09) | 0.48 (0.27-0.84) | 1,011 | 417 | 0.86 (0.75-1.00) | 0.85 (0.72-0.99) | 2,845 | 1,952 | 0.78 (0.71-0.85) | 0.83 (0.75-0.92) |
| Antipsychotics |  |  |  |  |  |  |  |  |  |  |  |  |  |  |  |  |
| No | 82,369 | 48,816 | Ref | Ref | 576 | 160 | Ref | Ref | 3,935 | 1,726 | Ref | Ref | 10,644 | 7,720 | Ref | Ref |
| Yes | 3,117 | 1,933 | 1.12 (1.04-1.21) | 1.22 (1.13-1.32) | 18 | 11 | 4.09 (1.56-10.72) | 11.57 (3.37-39.68) | 138 | 63 | 1.08 (0.76-1.51) | 1.21 (0.83-1.75) | 410 | 287 | 0.88 (0.71-1.10) | 1.10 (0.88-1.39) |
| Antidepressants |  |  |  |  |  |  |  |  |  |  |  |  |  |  |  |  |
| No | 70,313 | 41,901 | Ref | Ref | 512 | 151 | Ref | Ref | 3,288 | 1,457 | Ref | Ref | 8,781 | 6,456 | Ref | Ref |
| Yes | 15,173 | 8,848 | 0.95 (0.92-0.98) | 0.97 (0.94-1.01) | 82 | 20 | 0.77 (0.45-1.32) | 0.41 (0.20-0.85) | 785 | 332 | 0.92 (0.79-1.08) | 0.93 (0.78-1.12) | 2,273 | 1,551 | 0.77 (0.70-0.86) | 0.86 (0.77-0.96) |
| Statins |  |  |  |  |  |  |  |  |  |  |  |  |  |  |  |  |
| No | 59,792 | 35,005 | Ref | Ref | 431 | 116 | Ref | Ref | 2,927 | 1,252 | Ref | Ref | 7,807 | 5,630 | Ref | Ref |
| Yes | 25,694 | 15,744 | 1.12 (1.09-1.15) | 1.13 (1.09-1.17) | 163 | 55 | 1.38 (0.94-2.04) | 1.75 (1.02-3.00) | 1,146 | 537 | 1.18 (1.03-1.35) | 1.29 (1.10-1.51) | 3,247 | 2,377 | 1.06 (0.96-1.16) | 1.12 (1.00-1.24) |
| Non-steroid anti-inflammatory drugs | | |  |  |  |  |  |  |  |  |  |  |  |  |  |  |
| No | 57,481 | 34,445 | Ref | Ref | 429 | 118 | Ref | Ref | 2,809 | 1,220 | Ref | Ref | 8,181 | 5,932 | Ref | Ref |
| Yes | 28,005 | 16,304 | 0.93 (0.91-0.96) | 0.99 (0.96-1.02) | 165 | 53 | 1.25 (0.84-1.84) | 1.09 (0.69-1.72) | 1,264 | 569 | 1.07 (0.93-1.22) | 1.04 (0.90-1.20) | 2,873 | 2,075 | 0.99 (0.90-1.08) | 1.00 (0.91-1.11) |
| Antihypersentive  drugs |  |  |  |  |  |  |  |  |  |  |  |  |  |  |  |  |
| No | 38,466 | 22,588 | Ref | Ref | 293 | 84 | Ref | Ref | 1,898 | 829 | Ref | Ref | 4,909 | 3,593 | Ref | Ref |
| Yes | 47,020 | 28,161 | 1.05 (1.02-1.08) | 1.04 (1.01-1.08) | 301 | 87 | 1.01 (0.71-1.44) | 0.68 (0.42-1.10) | 2,175 | 960 | 1.02 (0.90-1.15) | 1.03 (0.89-1.19) | 6,145 | 4,414 | 0.93 (0.86-1.02) | 1.06 (0.96-1.17) |
| Opioids |  |  |  |  |  |  |  |  |  |  |  |  |  |  |  |  |
| No | 54,788 | 33,029 | Ref | Ref | 440 | 122 | Ref | Ref | 2,698 | 1,169 | Ref | Ref | 6,951 | 5,142 | Ref | Ref |
| Yes | 30,698 | 17,720 | 0.90 (0.87-0.93) | 0.87 (0.85-0.90) | 154 | 49 | 1.22 (0.82-1.81) | 1.35 (0.79-2.29) | 1,375 | 620 | 1.07 (0.94-1.22) | 1.14 (0.98-1.32) | 4,103 | 2,865 | 0.81 (0.75-0.89) | 0.97 (0.87-1.07) |
| Anti-thyroid drugs |  |  |  |  |  |  |  |  |  |  |  |  |  |  |  |  |
| No | 83,887 | 49,785 | Ref | Ref | 585 | 171 | Ref | Ref | 3,974 | 1,742 | Ref | Ref | 10,835 | 7,840 | Ref | Ref |
| Yes | 1,599 | 964 | 1.04 (0.94-1.15) | 0.98 (0.89-1.09) | 9 | 0 | N/A | N/A | 99 | 47 | 1.16 (0.78-1.73) | 1.17 (0.77-1.79) | 219 | 167 | 1.23 (0.90-1.68) | 1.28 (0.92-1.77) |
| Health care utilization in the year before index  date | | | | | | | | | | | | | | | | |
| Number of hospitalizations | | |  |  |  |  |  |  |  |  |  |  |  |  |  |  |
| 0 | 57,554 | 33,858 | Ref | Ref | 459 | 128 | Ref | Ref | 3,112 | 1,354 | Ref | Ref | 7,670 | 5,676 | Ref | Ref |
| 1 | 17,527 | 10,612 | 1.07 (1.04-1.11) | 1.17 (1.13-1.22) | 102 | 34 | 1.29 (0.82-2.05) | 1.22 (0.69-2.15) | 654 | 309 | 1.16 (0.98-1.38) | 1.27 (1.05-1.54) | 1,968 | 1,382 | 0.83 (0.74-0.92) | 0.98 (0.86-1.10) |
| >1 | 10,405 | 6,279 | 1.07 (1.02-1.11) | 1.29 (1.22-1.36) | 33 | 9 | 0.97 (0.44-2.14) | 1.44 (0.52-3.95) | 307 | 126 | 0.90 (0.71-1.15) | 1.08 (0.82-1.42) | 1,416 | 949 | 0.71 (0.63-0.81) | 0.95 (0.82-1.10) |
| Number of outpatient visits | | |  |  |  |  |  |  |  |  |  |  |  |  |  |  |
| 0 | 25,515 | 15,587 | Ref | Ref | 164 | 59 | Ref | Ref | 1,525 | 690 | Ref | Ref | 2,143 | 1,592 | Ref | Ref |
| 1 | 17,301 | 10,705 | 1.03 (0.99-1.08) | 0.94 (0.90-0.98) | 124 | 29 | 0.54 (0.32-0.92) | 0.41 (0.22-0.79) | 778 | 361 | 1.05 (0.88-1.25) | 1.05 (0.87-1.27) | 2,155 | 1,663 | 1.17 (1.02-1.35) | 1.15 (0.99-1.34) |
| >1 | 42,670 | 24,457 | 0.86 (0.83-0.88) | 0.84 (0.81-0.88) | 306 | 83 | 0.66 (0.44-0.99) | 0.46 (0.26-0.81) | 1,770 | 738 | 0.87 (0.75-0.99) | 0.90 (0.77-1.06) | 6,756 | 4,752 | 0.82 (0.74-0.92) | 0.96 (0.84-1.09) |
| Number of emergency room visits | | |  |  |  |  |  |  |  |  |  |  |  |  |  |  |
| 0 | 66,214 | 39,262 | Ref | Ref | 499 | 146 | Ref | Ref | 3,432 | 1,512 | Ref | Ref | 8,871 | 6,506 | Ref | Ref |
| 1 | 14,841 | 8,814 | 1.00 (0.97-1.04) | 0.96 (0.93-1.00) | 76 | 20 | 0.86 (0.50-1.49) | 0.56 (0.29-1.07) | 517 | 222 | 0.96 (0.79-1.15) | 0.86 (0.70-1.05) | 1,642 | 1,161 | 0.88 (0.78-0.99) | 1.00 (0.88-1.13) |
| >1 | 4,431 | 2,673 | 1.04 (0.98-1.11) | 1.02 (0.95-1.10) | 19 | 5 | 0.86 (0.31-2.44) | 0.74 (0.19-2.87) | 124 | 55 | 1.01 (0.71-1.45) | 0.99 (0.66-1.47) | 541 | 340 | 0.61 (0.51-0.74) | 0.80 (0.65-0.98) |

|  |  |
| --- | --- |
| **Exposure Drugs** | **ATC codes** |
| Alendronate | M05BA04 |
| Alendronate and colecalciferol | M05BB03 |
| Denosumab | M05BX04 |
| Etidronate | M05BA01 |
| Ibandronate | M05BA06 |
| Parathyroid hormone | H05AA03 |
| Raloxifene | G03XC01 |
| Risedronate | M05BA07 |
| Strontium ranelate | M05BX03 |
| Teriparatide | H05AA02 |
| Zoledronic acid | M05BA08 |
|  |  |
| **Concomitant drugs** | **ATC codes** |
| Oral corticosteroids | H02 |
| Anti-coagulants | B01AA, B01AE, B01AF, B01AB, B01AX, |
| Anti-diabetic drugs | A10 |
| Antiplatelet therapy (incl. Aspirin) | B01AC, N02BA01, N02BA51 |
| Hormone replacement therapy | G03C, G03D, G03F, L02AA |
| Hormone deprivation therapy | G03H, L02BA, L02BG |
| Anxiolytics and sedatives | N05B, N05C |
| Antipsychotics | N05A |
| Antidepressants | N06A |
| Statins | C10AA |
| Nonsteroidal anti-inflammatory drugs | M01A |
| Antihypertensive drugs | C02, C03, C04, C07, C08, C09 |
| Drugs for treatment of COPD | R03, R05, R07A |
| Opioids | N02A |
| Antithyroid drugs | H03B |
|  |  |
|  |  |
| **Comorbidities** | **ICD codes** |
| Chronic obstructive pulmonary disease | DJ44 |
| Chronic renal impairment | DN18 |
| Diabetes | DE10-DE14, DO24 (except DO244), DH360, DG632, DH360, DN083 |
| Dementia | DF00-DF03, DF051, DG30, DG231, DG311, DG318B, DG318E |
| Osteoporosis | DM80, DM81, DM82 |
| Malignancies, all | DC00-DC96 |
| Hip fracture | DS720–DS722 |
| Vertebral fracture | DS12, DS220, DS221, DS320, DT08 |
| Forearm fracture (radius and ulna) | DS52, DS62 |
| Humerus fracture | DS42 |
| DXA scan procedure | UXRE80, UXRE82, UXRE85, UXRF80, UXRF83, UXRG80, UXRG81, UXRG82A |
| Paget's disease | DM88 |
